# Allelopathic Effect of Quercetin, a Flavonoid from *Fagopyrum esculentum* Roots in the Radicle Growth of *Phelipanche ramosa*: Quercetin Natural and Semisynthetic Analogues Were Used for a Structure-Activity Relationship Investigation

**DOI:** 10.3390/plants10030543

**Published:** 2021-03-13

**Authors:** Mónica Fernández-Aparicio, Marco Masi, Alessio Cimmino, Susana Vilariño, Antonio Evidente

**Affiliations:** 1 Institute for Sustainable Agriculture, Spanish National Research Council (CSIC), 14004 Cordoba, Spain; 2 Department of Chemical Sciences, University of Naples Federico II, Complesso Univ. Monte Sant’Angelo, Via Cintia, 80126 Napoli, Italy; marco.masi@unina.it (M.M.); alessio.cimmino@unina.it (A.C.); 3 ALGOSUR S.A., Ctra Lebrija-Trebujena km 5.5, Lebrija, 41740 Sevilla, Spain; svilarino@algosur.com

**Keywords:** buckwheat, broomrape weeds, haustorium, allelopathic flavonoids, DMBQ, structure-activity relationship (SAR), sustainable crop protection

## Abstract

Allelopathic potential of buckwheat roots on the radicle growth of the broomrape weed species *Orobanche cumana* and *Phelipanche ramosa* was studied. Buckwheat root exudates induced a significant growth inhibition in *P. ramosa* radicles but radicles of *O. cumana* were not affected. Among the metabolites present in the root organic extract we identified the flavonol quercetin and the stilbene *p*-coumaric acid methyl ester with only quercetin showing inhibitory effect on *P. ramosa*. The activity of quercetin was compared with other two similar flavanoids, the flavone apigenin and the dihydroflavanol 3-*O*-acetylpadmatin extracted respectively from *Lavandula stoechas* and *Dittrichia viscosa* plants. In this comparative assay only 3-*O*-acetylpadmatin besides quercetin, showed inhibition activity of radicle growth while apigenin was inactive. These results indicated that the presence of two *ortho*-free hydroxy groups of C ring, like catechol, could be an important feature to impart activity while the carbon skeleton of B ring and substituents of both A and B rings are not essential. Besides reduction of radicle growth, haustorium induction was observed at the tip of *P. ramosa* radicles treated with quercetin which swelled and a layer of papillae was formed. Activity of quercetin on haustorium induction in *P. ramosa* was assayed in comparison with the known haustorium-inducing factor 2,6-dimethoxy-*p*-benzoquinone (DMBQ) and a three partial methyl ether derivatives semisynthetized from quercetin. Results indicated that *P. ramosa* haustorium was induced by DMBQ at concentrations of 1–0.5 mM and quercetin and its derivatives at concentration range 0.1–0.05 mM.

## 1. Introduction

The parasitic broomrape weeds (*Orobanche* and *Phelipanche* species) have not functional roots nor photosynthetic activity obtaining all nutrients and water from the crop root by haustorial connections with the vascular system [1]. Their expansion in Mediterranean Basin and Asia is uncontrolled infecting crops in the Apiaceae, Asteraceae, Brassicaceae, Fabaceae or Solanaceae becoming a threat to food security [1,2]. Broomrapes are one of the most difficult-to-control of all weeds, because the difficult application of methods that can kill the broomrapes without damaging the crop to which they are physically and biochemically overlapped through the haustorium. Additional factors challenging broomrape control is their high fecundity, persistent seedbank, and rapid responses to changes in agricultural practices, adapting to new hosts with increased aggressiveness [3].

Weed management is essential for agricultural production, and it has relayed on traditional chemical herbicides for decades with efficient control but carrying the long-term problems of agroecosystem contamination, undesirable health effects and the emergence of herbicide-resistant weed populations [4]. Due to the undesirable effects of herbicides, the number of old herbicides authorized are in constant decline with few novel herbicides in development [5,6] which prompt the need for the development of novel nature-inspired bioherbicides containing either microbial strains or toxins from microbial or plant origin. From the screening of toxins of microbial and plant origins new compounds with antagonistic activity against parasitic weeds have been discovered [7,8,9,10,11,12,13,14,15]. In many cases the natural herbicidal substances lack appropriate physicochemical properties for field application and their use in agriculture depends on the development of formulations that increase the solubility in water [16,17] or the development of strategies for the application of the whole organic extract of allelopathic plants or incorporating them into biofilms [18].

Buckwheat (*Fagopyrum esculentum* Moench) is a short life cycle crop from the Polygonaceae with activity in weed suppression [19,20]. Despite being the subject of many studies there is no conclusive evidence of which compounds are responsible for the allelopathic suppression of weed growth, although it has been suggested that phenolic acids and flavonoids could be responsible [21,22]. Buckwheat roots exudate to the rhizosphere allelochemicals with inhibitory effect on weeds mainly palmitic and gallic acid [23,24]. The roots extracts of buckwheat contain allelopathic flavonoids mainly catechin, and isoquercitrin [22]. To the best of our knowledge, there are currently no reports on the effects of buckwheat on parasitic weeds. From a previous field screening of the USDA buckwheat germplasm collection, the buckwheat accession PI 658422 from Nepal was selected in our lab for allelopathic activity. This manuscript reports the allelopathic activity of roots of buckwheat accession PI 658422 on the radicle growth of *Phelipanche ramosa* and the isolation and identification of the flavanol quercetin with inhibitory activity. The activity of quercetin was compared with similar flavonoids previously isolated in our laboratory from different plant origins, i.e., the flavone apigenin and the dihydroflavanol 3-*O*-acetylpadmatin extracted, respectively, from *Lavandula stoechas* [25] and *Dittrichia viscosa* [26]. In addition, quercetin activity was compared with that of semisynthetic methyl ether derivatives of quercetin to elucidate structure activity relationships (SAR).

## 2. Results and Discussion

**Figure 1:**
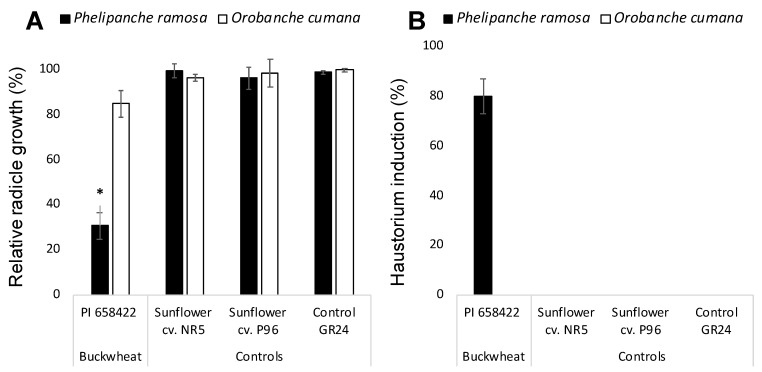
Allelopathic effect of hydroponically collected buckwheat and sunflower root exudates on growth (**A**) and haustorium formation (**B**) in radicles of *Phelipanche ramosa* and *Orobanche cumana*. * Indicates differences at the 0.05 level compared with the control GR24. Error bars represent the standard error of the mean.

Allelopathic potential of buckwheat root exudates was assayed on radicle development of two broomrape species *P. ramosa* and *O. cumana* and the effect of buckwheat compared with the effect of two sunflower cultivars NR5 and P96 and the negative control GR24. Broomrape seeds only germinate upon detection of germination stimulants exuded by host roots. For allelopathic bioassays, broomrape seeds require the induction of germination with the synthetic germination stimulant GR24 active both in *O. cumana* and *P. ramosa* that act as a negative control for radicle growth inhibition [12,14]. Growth of *O. cumana* radicles treated with a combination of buckwheat root exudates and GR24 was not significantly different from the growth of GR24-treated *O. cumana* control radicles nor the growth of radicles treated with combination of sunflower root exudates and GR24. However, *P. ramosa* seeds treated with the combination of buckwheat root exudate and GR24 displayed shorter radicles with a swelled tip and a layer of papillae in its surface which indicates that haustorium was formed. The radicle growth cessation and haustorium formation was not observed in *P. ramosa* radicles when treated with the negative control GR24 nor when treated with the combination of sunflower root exudates and GR24 (Figure 1A,B).

**Figure 2:**
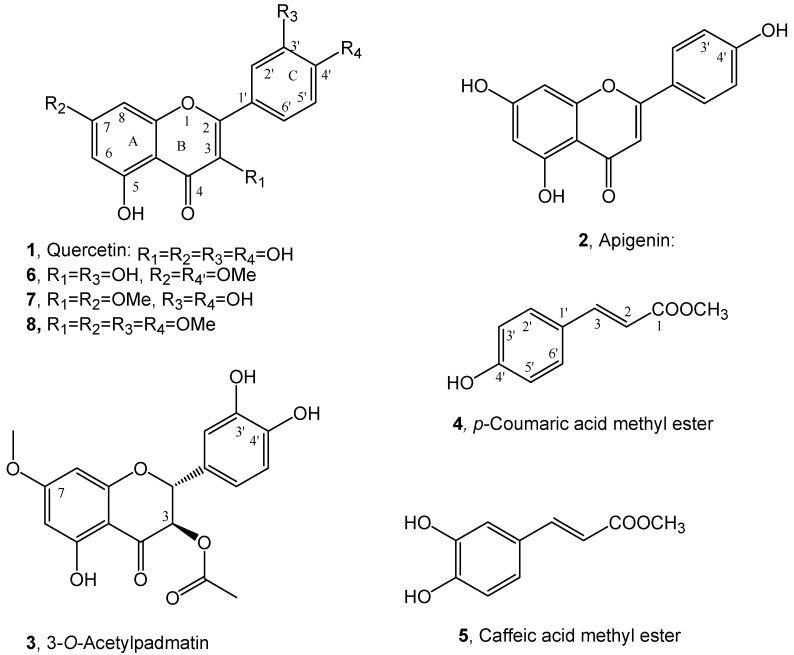
Structure of quercetin, apigenin and 3-*O*-acetylpadmatin (**1**–**3**), *p*-coumaric and caffeic acid methyl esters (**4** and **5**) and the two dimethyl (**6** and **7**) and tetramethyl (**8**) derivatives of quercetin.

The purification of the organic extract obtained from dried roots of buckwheat (*Fagopyrum esculentum*) by combined column and TLC chromatography, as detailed reported in the Materials and Methods section, afforded quercetin and the methyl ester of *p*-coumaric acid (**1** and **4**, Figure 2). They were characterized by comparison of their spectroscopic data (essentially ^1^H NMR and ESI MS) with those reported in literature for **1** by Grande at al. [26] and for **4** by Karthikeyan et al. [27] and by data reported in Materials and Methods section and SI. Compounds **1** and **4** belong to cynnamic acids and flavonols groups of natural occurring substances and are both biosynthesized via shikimic acid pathway [28]. They are already reported as plant [29,30,31] and fungi [32] bioactive metabolites. Although quercetin is found in many vegetables, it is present in low amounts in Polygonaceae family [22]. Golisz et al. [33] identified quercetin in buckwheat among eight allelochemicals including rutin, (+)-catechin, (−)-epicatechin, chlorogenic acid, caffeic acid, ferulic acid, and gallic acid. The presence of quercetin is low in aerial vegetative organs of buckwheat [33] but its concentration increases in leaves at plant maturity [22]. The content of quercetin in buckwheat roots has been described as low and only detected at buckwheat flowering stage [22]. Previous studies of buckwheat root deposits in soil did not detect quercetin while it was detected in agar plates after buckwheat germination [24]. Several studies indicate that there is a wide variation in contents of allelopathic flavonoids depending on the variety, phenological stage, and environmental conditions [34,35,36,37,38,39,40,41]. Levels of quercetin in buckwheat increase in drier [37] and sunnier [35] weathers. Recently *p*-coumaric acid methyl ester and its close analogue methyl ester of caffeic acid (**5**, Figure 2) were isolated together with two new copaane sesquiterpenoids, named stoechanones A and B, from *Lavandula stoechas* whose organic extract showed strong herbicidal activity against the noxious weed *Amarantus retroflexus* [25]. Caffeic acid methyl ester (**5**) was identified comparing its spectroscopic data (essentially ^1^H NMR and ESI MS) with those reported in literature [42]. From the same plant organic extract also apigenin (**2**, Figure 2), a flavanone which belongs to another subgroup of flavonoids and thus close to **1** was isolated and identified by comparing its spectroscopic data (essentially ^1^H NMR and MS) with those reported in literature [43] (see Materials and Methods section and SI). Independently working on *Dittrichia viscosa,* as potential allelopathic plant, four new phytotoxic sesquiterpenoins, named inuloxins A-D and α-costic acid were firstly isolated [15] and successively also 3-*O-*acetylpadmatin (**3**, Figure 2), a dihydroflavonol which belongs to another subgroup of flavonoids and thus close to **1** and **2**. Compound **3** was identified by comparing its physic and spectroscopic (essentially specific optical rotation and ^1^H and ^13^C NMR and ESI MS) data with those reported in literature [26]. In particular, the correlations observed in the HMBC NMR spectrum were essential to assign the oxygenated and not quaternary sp^2^ carbons [44] (see Materials and Methods and SI).

**Figure 3:**
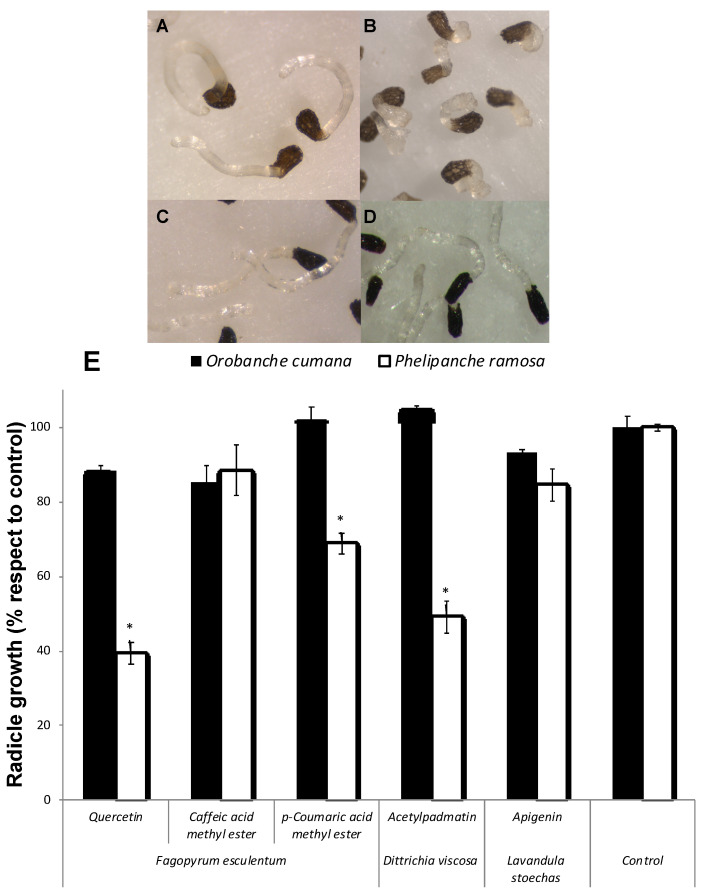
Inhibition of radicle growth of broomrape species. *P. ramosa* radicles treated with control (**A**) and with quercetin (**B**); *O. cumana* radicles treated with control (**C**) and quercetin (**D**). Allelopathic effect of quercetin, caffeic acid and *p*-coumaric acid methyl esters, apigenin and acetylpadmatin on radicle growth of *P. ramosa* and *O. cumana* (**E**). * Indicates differences at the 0.05 level compared with the control. Error bars represent the standard error of the mean.

Allelopathic effects of the flavonoids quercetin, apigenin and 3-*O-*acetylpadmatin (**1**-**3**), and caffeic acid and coumaric acid methyl esters were assayed on *P. ramosa* and *O. cumana* seedlings (Figure 3). Quercetin (**1**) showed a strong inhibition of the radicle growth of *P. ramosa* seedlings in comparison with the control (Figure 3A,B,E). Flavonoids have well known growth inhibitory activities being frequently involved in root allelopathy. Golisz et al. [33] observed the quercetin inhibitory activity of root growth in lettuce seedlings. Inhibition of *P. ramosa* radicle growth was also induced by acetylpadmatin (**3**) while apigenin (**2**) and caffeic acid were inactive (Figure 3E). The allelopathic effect of **1** and **3** in *P. ramosa* radicles was associated with a cessation of the radicle elongation and not associated with darkening or any other visible sign of toxicity in the radicle tissue. In addition, the tip of the *P. ramosa* radicles treated with **1** and **3** became swallowed into a spherical form and differentiated the haustorial organ while *P. ramosa* radicles treated with the rest of the compounds or with the control did not. A slight but significant reduction in radicle growth without haustorium formation was observed in *p*-coumaric-treated *P. ramosa* radicles. Quercetin had no allelopathic effect on *O. cumana* radicle in comparison with the control (Figure 3C–E), nor the rest of the compounds tested (Figure 3E). Species-specific differences in allelopathic effects have been described for species of *Orobanche* and *Phelipanche* genera. They have different host ranges, and they differ in their capacity for signal perception and sensibility to inhibition by allelochemicals [8,9,12,14].

Quercetin is the most widely distributed flavone in the plant kingdom playing several roles in the rhizosphere during plant-microbial associations [45]. Quercetin was reported with stimulatory activity on the hyphal growth of *Glomus margarita* and the authors hypothesized that the hydroxyl group in position 3 is essential to confer stimulatory activity, and concluded, that flavonols in general should be more stimulatory than flavones. Among the flavonols tested by these authors, quercetin, with hydroxyl groups on positions 3′ and 4′, gave the greatest stimulation of hyphal growth. Quercetin-3-*O*-galactoside was found to be the dominant flavonoid released from alfalfa seeds promoting spore germination of *G. etunicatum* and *G. macrocarpum* [46]. Quercetin also enhances nodulation by *Rhizobium etli* or *R. tropici* in bean roots [47]. Other activities of quercetin during plant-microbe associations have also been described such the induction of resistance against *Pseudomonas syringae* by increasing H_2_O_2_ and callose production [48]. Reactive oxygen species generation is indispensable for haustorium formation in *Striga*, being observed a strong accumulation of H_2_O_2_ [49]. Quercetin induced haustoria in *Triphysaria versicolor* roots [50]. The effect of **1** and **3** in *P. ramosa* radicles may be associated with the presence of the two *ortho*-free hydroxy group of C ring, like catechol, are an important feature to impart activity and the carbon skeleton of B ring and substituents on both A and B ring are not essential. The result does not surprise as the presence of two *ortho*-hydroxy phenolic groups (like catechol) as well as that of two *para* hydroxy phenolic group (as hydroquinone) represents of an oxidoreductive couples. The relation between the quinone/hydroquinone skeleton and its biological activity is already known. In fact, in previous structure activity relationships study using sphaeropsidone and *epi*sphaerosidone and some of their derivatives testing their ability to initiate haustorium development in *Striga* and *Orobanche* species was demonstrated that the conversion of the natural sphaeropsidones, their analogues, and hemisynthetic derivatives in the corresponding 3-methoxyquinone and this finally, by reductive opening of the epoxy group followed by water nucleophilic elimination into the 3-methoxyquinone, is fundamental to impart activity [51]. This hypothesis is in full agreement with the activity observed by quinones as sorghum xenognosin and dimethoxybenzoquinones. The latter is very close to the 3-methoxyquinone, which as above explained could be generated from oxidation of sphaeropsidone, could play a role in the chemistry in host recognition parasitic angiosperms. Thus, quinone/hydroquinone structures serve as cofactors in many metabolic pathways, playing critical chemical roles in oxidation/reduction processes [28,52,53]. This mode of action could also operate in the haustorium-induction in broomrape [51]. Similar structure activity relationships were also observed in additional studies carried out by assaying *epi*-epoformin, a phytotoxic cyclohexene epoxide isolated from the *Diplodia quercivora*, responsible *Quercus canariensis* declining in Tunisia [54], and some of its semisynthetic derivatives in an etiolated wheat coleoptile bioassay [55]. In addition, the importance of the quinone/hydroquinone skeleton was also recently observed testing the phytotoxicity, on host and non-host plants of three new anthraquinones, named lentiquinone A-C and the already known lentisone, pachybasin, ω-hydroxypachybasin, 1,7-dihydroxy-3-methylanthracene- 9,10-dione, and phomarin isolated from *Ascochyta lentis*, the causal agent of ascochyta blight on lentil [56,57].

To confirm these SAR results three methyl derivatives of quercetin were prepared from **1** by reaction with diazomethane. The crude reaction mixture was purified as detailed reported in the Materials and Methods section and the main derivatives isolated were the 7,4′-*O*,*O*′- and 3,7-*O*,*O*′- dimethyl (**6** and **7**, Figure 2) and the 3,7,3′,4′-*O*,*O*′,*O*″, O‴-tetramethyl (**8**, Figure 2) derivatives of quercetin. The ^1^H NMR and ESI data of **6** and **7** were reported in Materials and Methods and SI. These data agree with those previously reported for **6** by Haraguchi et al., [58] and for **7** by Valesi et al., [59]. The unambiguously location of the methoxy groups at C-7 and C-4′ and C-3 and C-7 in **6** and **7,** respectively, was obtained recording their NOESY spectra [60] (see SI). The ^1^H NMR and ESI MS data of **8** are reported in Materials and Methods and SI and are in agreement to those previously reported [59].

**Figure 4:**
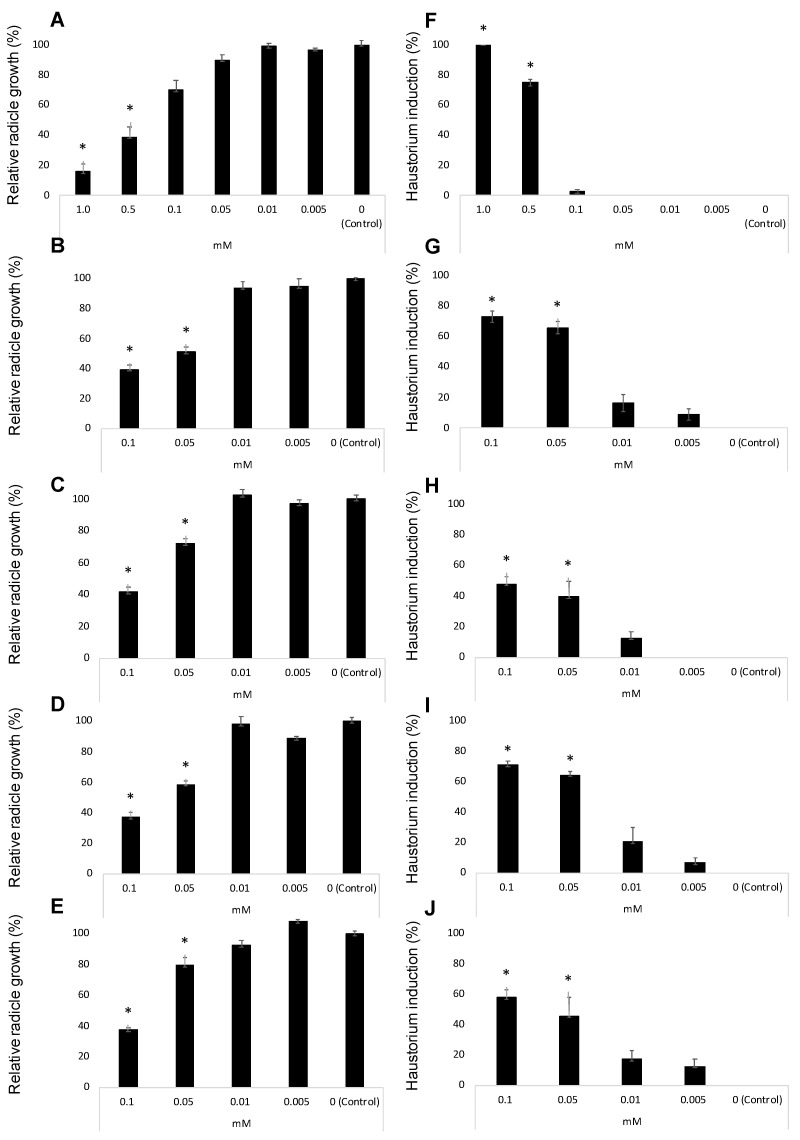
Allelopathic effects of DMBQ (**A**,**F**), quercetin (**B**,**G**), 7,4′-*O*,*O*′-dimethylquercetin (**6**) (**C**,**H**), 3,7-*O*,*O*′-dimethylquercetin (**7**) (**D**,**I**), and 3,7,3′,4′-*O*,*O*′,*O*″,*O*‴-tetramethylquercetin (**8**) (**E**,**J**) in the radicle growth (**A**–**E**) and haustorium induction (**F**–**J**) of *Phelipanche ramosa*. * Indicates differences at the 0.05 level compared with the control. Error bars represent the standard error of the mean.

Figure 4A–E illustrate the inhibition of radicle growth and Figure 4F–J the induction of haustorium development observed during the evaluation of the activity of the three methyl derivatives of quercetin. Their activity was compared with the activity of quercetin and 2,6-dimethoxy-*p*-benzoquinone (DMBQ) the strongest haustorium-inducing factor active in radicles of other parasitic plants such as *Striga* spp. and *Triphysaria* spp. DMBQ is active inducing *Triphysaria* haustorium between 1 and 30 μM concentrations. At concentrations of 100 μM or higher DMBQ is toxic to *Triphysaria* roots [52]. In *Striga* species, the active range spans from 0.05 to 10 μM being toxic at 50 μM or higher [49,61]. Unlike *Striga* spp. and *Triphysaria* spp., broomrape species has been reported to do not respond to DMBQ with haustorium initiation [62,63,64,65] but the activity has been tested only at 10 μM [65] and the activity at higher concentrations has not been reported.

**Figure 5:**
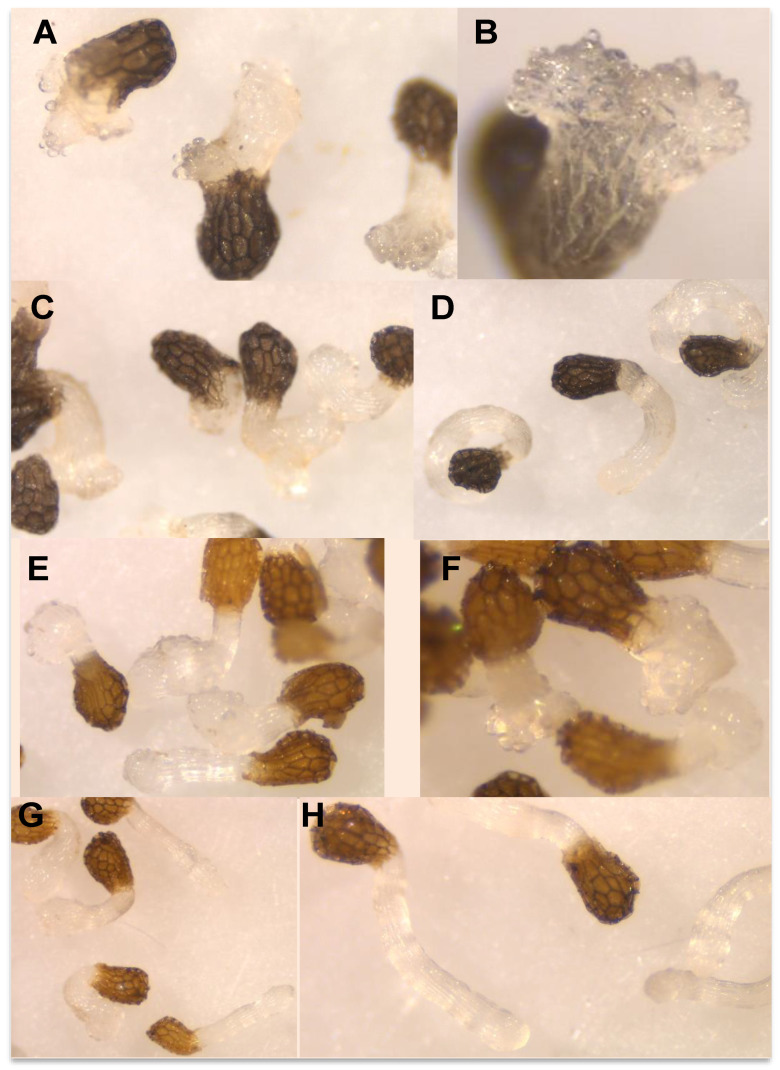
Effects in *P. ramosa* radicle of (**A**) DMBQ 1 mM; (**B**) DMBQ 1 mM detail; (**C**) DMBQ 0.5 mM; (**D**) DMBQ 0.1 mM; (**E**) 7,4′-*O*,*O*′-dimethylquercetin (**6**) 0.1 mM; (**F**) 3,7-*O*,*O*′-dimethylquercetin (**7**) 0.1 mM; (**G**) 3,7,3′,4′-*O*,*O*′,*O*″,*O*‴-tetramethylquercetin (**8**) 0.1 mM; (**H**) control.

In our work, DMBQ induced the cessation of *P. ramosa* radicles growth and a swelling of *P. ramosa* tip with a formation of a papillae layer (Figure 5A–C). Figure 4A shows the radicle growth inhibition and Figure 4F the proportion of radicles that developed haustorium when *P. ramosa* was treated with DMBQ at a range of 1 mM to 5 μM showing that the active range for haustorium induction is found at concentrations of 0.5 mM or higher. Our results indicate that in *P. ramosa* radicles treated with DMBQ at 1 mM the radicle growth was only 16% of that of the control (Figure 4A, Figure 5A,B,H) with haustorium visible in 100% of the radicles (Figure 4F, Figure 5A,B) while in *P. ramosa* radicles treated with DMBQ at 0.5 mM the radicle growth was 39% of that of the control (Figure 4A, Figure 5H) with haustorium visible in 75% of the radicles (Figure 4F, Figure 5C). The haustorium-inducing effect of DMBQ on *P. ramosa* radicles disappeared at 100 μM and at lower concentrations (Figure 4F and Figure 5D). DMBQ did not induce visible signs of toxicity in *P. ramosa* as has been observed in other parasitic weeds which have been described as turning brown and die at concentrations higher than 50 μM [52,61]. Unlike more hydrophobic quinones, DMBQ is sufficiently soluble in water to make fresh working stocks at 1 mM directly in water, without the solvent DMSO usually used in labs to make stock solutions for haustorial induction assays of *Phelipanche*, *Striga*, and *Triphysaria* [49,65,66]. The effect of quercetin was evaluated at concentration range of 100 μM and 5 μM (Figure 4B,G). No visible signs of toxicity were observed in *P. ramosa* radicles. Quercetin induced haustorium in *P. ramosa* radicles at concentrations of 50 μM or higher. At 50 μM the average of radicle growth was only 51% of that of the control and 66% of the radicles carried haustorium. Among the three methyl derivatives evaluated, 3,7-*O*,*O*′-dimethylquercetin (**7**) showed the highest activity in the radicle growth inhibition and haustorium induction tests (Figure 4D,I). The growth inhibition at 50 μM by 7,4′-*O*,*O*′-dimethylquercetin (**6**) and 50 μM 3,7,3′,4′-*O*,*O*′,*O*″,*O*‴-tetramethylquercetin (**8**) was slightly reduced in comparison with quercetin and compound **7** as shown in Figure 4C,E. Furthermore, derivatives **6** and **8** induced lower haustorium development in comparison to quercetin and 3,7-*O*,*O*′-dimethylquercetin (**7**) (Figure 4H,J). Perception of haustorium-inducing factors promotes a cessation of parasite root growth with a rapid swelling developing an adhesive structure that attaches the parasite to the host surface from which the invasive organ subsequently develops [3]. In parasitic weeds such as *Triphysaria* and *Striga* several haustorium-inducing factors have been identified including phenolics, flavonoids, and *p*-benzoquinones with different concentrations and times of exposure required for optimal haustorium induction [67]. The length of the root that develops haustorium may depend on the strength and the concentration of the haustorium-inducing factor and the timing of detection. These processes have not been well characterized for broomrape species and until recently, it was believed to not being initiated by haustorium-inducing chemical agents [51,62,63,64,65]. Some authors observed the activity of quercetin in root growth inhibition without activity in cytodifferentiation [33]. Others, observed haustorium differentiation activity in quercetin [50]. In this work, we have characterized the effect of quercetin and its derivatives independently in *P. ramosa* radicle length and rate of haustorium induction since we cannot rule out the effect of quercetin acting specifically on root growth in addition to its haustorium-inducing effect.

## 3. Materials and Methods

### 3.1. General Experimental Procedures

Optical rotations were measured in MeOH on a P-1010 digital polarimeter (Jasco, Tokyo, Japan), ^1^H and ^13^C NMR spectra were recorded at 500 and 400 and 125 and 1000 MHz in on Varian (Palo Alto, CA, USA) and Bruker (Karlsruhe, Germany). The same solvent was used as internal standard. The multiplicities were determined by DEPT spectrum [60] COSY, HSQC, HMBC and NOESY spectra were recorded using Bruker microprograms. ESI MS spectra were recorded on a 6120 Quadrupole LC/MS instruments (Agilent Technologies, Milan, Italy), respectively. Analytical and preparative TLC were performed on silica gel (Kieselgel 60, F_254_, 0.25 and 0.5 mm respectively) and on reversed phase (Kieselgel 60 RP-18, F_254_, 0.20 mm) plates (Merck, Darmstadt, Germany). The spots were visualized by exposure to UV radiation (253 nm), or by spraying first with 10% H_2_SO_4_ in MeOH and then with 5% phosphomolybdic acid in EtOH, followed by heating at 110 °C for 10 min. Column chromatography was performed using silica gel (Merck, Kieselgel 60, 0.063–0.200 mm). Quercetin and caffeic acid were purchased from Sigma-Aldrich Milano, Italy)

### 3.2. Plant Material and Growth Conditions

Buckwheat (*Fagopyrum esculentum*) roots and root exudates were obtained from the buckwheat accession PI 658422 collected in Nepal and kindly provided by USDA. Buckwheat seeds were surface sterilized with 4% sodium hypochlorite containing 0.02% Tween 20, rinsed three times with sterile distilled water and placed on moistened filter paper inside Petri dishes to allow germination. Four days later, germinated buckwheat seeds were transferred to pots filled with sterile perlite in a growth chamber (23/20 °C, 16/8 h day/night). Plants received Hoagland’s nutrient solution modified at one-quarter strength twice per week. For collection of roots, buckwheat plants were removed from the perlite, roots were carefully washed in distilled water, quickly dried with filter paper, immediately frozen and maintained at −80 °C until lyophilization. For determination of buckwheat allelopathic activity on *Orobanche cumana* and *Phelipanche ramosa* radicle growth, root exudates were collected from buckwheat accession PI 658422 and two sunflower control cultivars NR5 and P96. Three plants of each cultivar were grown as described above, removed from the perlite, their roots carefully washed and individually placed in tubes immersing the roots in sterile distilled water. After 24 h, the solutions containing the buckwheat and sunflower root exudates were collected and the total crop root contained in each tube weighed. Root exudate solution was adjusted with sterile distilled water to achieve equivalent concentrations of 0.02 g of crop root fresh weight /mL of hydroponic media (root exudate solution) and tested for allelopathic potential as described in Section 3.4 below. Fresh bunches of *Lavandula stoechas* in the flowering stage were purchased from the vegetable market in Algiers and a specimen was deposited at Ecole Nationale Supérieure d’Agronomie, ENSA, Algeria. Whole aerial parts of *Dittrichia viscosa* plant were collected fresh in Italy and Algeria from naturally occurring populations. A voucher specimen was deposited at the herbarium of the Ecole National Supérieure Agronomique in Algiers. After harvesting, leaves were detached from the stems and dried in a ventilated oven at 50 °C for two days. Seeds of two broomrape species: *O. cumana*, population collected in sunflower in Spain and *P. ramosa,* population collected in oilseed rape in France were used to determine allelopathic potential of tested metabolites.

### 3.3. Extraction Purification and Identification of Buckwheat Metabolites

Dried roots of buckwheat (6.2 g) were minced in a Blender mill and macerates overnight at dark in H_2_O-MeOH (1:1, 150 mL). The mixture was centrifuged at 7000 r.p.m. and the alcoholic-aqueous phase extracted first with *n*-hexane (3 × 150 mL) and then with CH_2_Cl_2_ (3 × 150 mL). The CH_2_Cl_2_ organic extracts were combined, dried (Na_2_SO_4_) and concentrated under vacuum to yield on oily brown residue (41.5 mg). This latter was fractionated by TLC, eluted with CHCl_3_-EtOAc-MeOH 6:2:2, affording a pure homogeneous solid identified as below reported as quercetin (**1**, 15 mg). In another experiment, the same extraction procedure was applied to buckwheat dried roots (5.5 g) yielding the CH_2_Cl_2_ extract as an oil. The latter was purified by TLC but eluted with the different solvent system CHCl_3_-EtOAc-MeOH 6:3:1, affording a homogeneous solid (**4**, 2.3 mg) identified as methyl ester of *p*-coumaric acid.

*Quercetin (2-(3,4-dihydroxyphenyl)-3,5,7-trihydroxy-4H-chromen-4-one) (**1**)*: ^1^H NMR, (CD_3_OD, 400 Mz) δ, 7.75 (1H, d, *J* = 2.1 Hz, H-2′), 7.63 (1H, dd, *J* = 8.5 and 2.1 Hz, H-6′), 6.90 (1H, d, *J* = 8.5 Hz H-5′), 6.41 (1H, d, *J* = 2.0 Hz, H-8), 6.20 (1H, d, *J* = 2.0 Hz H-6) these data are very similar to those previously recorded at 60 MHz in acetone-_d6_ [26]; ESI MS, *m/z* 303 [M + H]^+^.

p-*Coumaric acid methyl ((*E*)-methyl 3-(4-hydroxyphenyl)acrylate) (**4**)*: ^1^H NMR (CDCl_3_, 500 MHz), δ, 7.67 (1H, d, *J* = 15.5 Hz, H-3), 7.46 (2H, d, *J* = 8.6 Hz, H-2′6′),) 6.88 (2H, d, *J* = 8.6 Hz, H-3′,5′), 6.33 (1H, d, *J* = 15.5 Hz, H-2), 3.84 (3H, s, OMe); these data are very similar to those previously recorded in CD_3_OD [27]; ESI MS, *m/z* 179 [M + H]^+^.

### 3.4. Apigenin and Methyl Ester of Caffeic from Lavandula stoechas

Dried *L. stoechas* plants (200 g) was minced in Blender mill and macerated overnight with MeOH-H_2_O (1:1, 1 L) and the alcoholic-aqueous extract than centrifuged and exhaustively extracted with CH_2_Cl_2_ as previously reported [25]. The organic extract was purified by combined column and TLC chromatography to afford apigenin as a yellow solid (**2**, 4.6 mg) and the methyl ester of caffeic acid (5, 5.2 mg).

*Apigenin (4′,5,7-trihydroxyflavone) (**2**):*^1^H NMR (CD_3_OD, 400 MHz), δ, 7.87 (2H, d, *J* = 8.9 Hz, H-2′,6′), 6.95 (2H, d, *J* = 8.9 Hz, H-3′,5′), 6.61 (1H, s, H-3), 6.46 (1H, d, *J* = 2.1 Hz, H-8), 6.22 (1H, d, *J* = 2.1 Hz, H-6); these data are very similar to those previously recorded in(CD_3_)_2_SO [43]; ESI MS, *m/z* 271 [M + H]^+^.

*Methyl ester of caffeic acid ((*E*)-methyl 3-(3,4-dihydroxyphenyl)acrylate) (**6**):*^1^H NMR (CD_3_OD, 500 MHz), δ, 7.56 (1H, d, *J* = 16.1 Hz, H-3), 7.05 (1H, br s, H-2′), 6.96 (1H, d, *J* = 8.2 Hz, H-5′), 6.79 (1H, d, *J* = 8.2 Hz, H-6′), 6.27 (1H, d, *J* = 16.1 Hz, H-2), 3.77 (3H, s, OMe) these data are very similar to those previously reported [42]; ESI MS, *m/z* 216 [M + Na]^+^, 195 [M + H]^+^, 163 [M − CH_3_OH]^+^.

### 3.5. 3-O-Acetylpadmatin from Dittrichia viscosa

Dried *D. viscosa* plant was minced in Blender mill and macerated overnight with MeOH-H_2_O (1:1, 1 L) and the alcoholic-aqueous extract than centrifuged and exhaustively extracted first with *n*-hexane and then with extracted with CH_2_Cl_2_ as previously reported [15]. The organic extract was purified by combined column and TLC chromatography to afford a homogeneous compound identified as below reported 3-*O*-acetylpadmatin (**3**, 20 mg).

*3-*O*-Acetylpadmatin (Acetic acid 2-(3,4-dihydroxy-phenyl)-5-hydroxy-7-methoxy-4-oxo-chroman-3-yl ester) (**3**)*: [α]^25^_D_ +40.0 (*c* 0.6) (lit. 26: [α]_D_ +41.0 (*c* 0.84, MeOH)); ^1^H NMR (CDCl_3_, 400 MHz), δ, 11.46 (1H, br s, HO-5), 7.02 (1H, br s, H-2′), 6.87 (2H, br s, H-5′,6′), 6.10 (1H, br s, H-8), 6.04 (1H, br s, H-6), 5.83 (1H, d, *J* = 12.7 Hz, H-3), 5.22 (1H, d, *J* = 12.7 Hz, H-2), 3.81 3H, s, OMe), 2.05 (3H, s, MeCO) these data are very similar to those previously recorded at 200 MHz [26]; ESI MS *m/z*: 743 [2M + Na]^+^, 361 [M + Na]^+^.

### 3.6. Methylation of Quercetin

To quercetin (**1**, 30 mg) dissolved in MeOH (1 mL) was added an ethereal solution of diazomethane until yellow persisting color. After 2 h the reaction was stopped by evaporation under N_2_ stream. The residue was purified by TLC eluted with CHCl_3_-*iso*-PrOH (95:5) and three main derivatives were obtained. They are a tetramethyl- (**8**, 3.5 mg) and two dimethyl-quercetin derivatives (**6** and **7**, 6.6 and 3.6 mg, respectively).

*7,4′-*O,O′*-Dimethylquercetin (**6**)*: ^1^H NMR (CD_3_OD, 500 MHz), δ, 7.71 (2H, br s, *J* = 2.1, Hz, H-2′,6′), 7.08 (1H, d, *J* = 8.0 Hz, H-5′), 6.62 (1H, brs, H-6), 6.33 (1H, br s = H-8), 3.96 (3H, s, 4′-OMe), 3.95 (3H, s, 7-OMe), 3.87 (3H, s OMe) these data are very similar to those previously recorded in CCl_4_ [58]; ESI MS *m/z*: 353 [M + Na]^+^.

*3,7-*O,O′*-Dimethylquercetin (**7**)*: ^1^H NMR (CD_3_OD, 500 MHz), δ, 7.67 (1H, d, *J* = 2.1, Hz, H-2′), 7.58 (1H, dd, *J* = 8.5 and 2.1 Hz, H-6′), 6.92 (1H, d, *J* = 8.5 Hz, H-5′), 6.61 (1H, d, *J* = 2.2, H-6), 6.35 (1H, d, *J* = 2.2, H-8), 3.90 (3H, s, 7-OMe), 3.82 (3H, s, 3-OMe), 3.87 (3H, s OMe); these data are very similar to those previously recorded in CCl_4_ [59]; ESI MS *m/z*: 353 [M + Na]^+^.

*3,7,3′,4′-*O,O′,O′,O‴*-Trimethylquercetin (**8**):*^1^H NMR (CDCl_3_, 500 MHz), δ, 12.60 (1H, br s, HO-5), 7.74 (1H, dd, *J* = 8.6 and 2.7 Hz, H-6′), 7.70 (1H, d, *J* = 2.7, Hz, H-2′), 7.0 (1H, d, *J* = 8.6 Hz, H-5′), 6.46 (1H, d, *J* = 2.2, H-6), 6.36 (1H, d, *J* = 2.2, H-8), 3.97(6H, s, 2 x OMe), 3.88 (3H, s, MeO), 3.87 (3H, s OMe) these data are very similar to those previously recorded in CCl_4_ [59]; ESI MS *m/z*: 381 [M + Na]^+^.

### 3.7. Bioassay for Radicle Growth and Haustorium Induction

Allelopathic effects in radicle growth and haustorium induction by buckwheat and sunflower root exudates (0.02 g of root fresh weight /mL of root exudate solution), DMBQ (1 mM to 0.005 mM) and each of the following metabolites quercetin, apigenin, 3-*O*-acetylpadmatin (**1**–**3**), caffeic acid and *p*-coumaric acid methyl esters and the derivatives of quercetin 7,4′-O,O′- and 3,7-*O*,*O*′- dimethyl (**6** and **7**) and the 3,7,3′,4′-*O*,*O*′,*O*″,*O*‴-tetramethyl (8) (0.1 mM to 0.005 mM) was determined according to previous protocols [12,51,68,69]. Germination of broomrape seeds is achieved in the laboratory through a two-step process, a warm stratification called conditioning followed by an induction of germination by the synthetic strigolactone GR24 [70]. Broomrape seeds were surface sterilized by immersion in 0.5% (*w*/*v*) NaOCl and 0.02% (*v*/*v*) Tween 20, for 5 min, rinsed thoroughly with sterile distilled water, and dried in a laminar air flow cabinet. Approximately 100 seeds of each broomrape species were placed separately in 9 mm diameter glass fiber filter paper disks (GFFP) (Whatman International Ltd., Maidstone, UK) moistened with 50 μL of sterile distilled water and placed inside Petri dishes in incubators at 23 °C during 10 days to allow seed conditioning. GFFP disks containing conditioned broomrape seeds were transferred onto a sterile sheet of filter paper to remove the excess of water and transferred to new 10 cm sterile Petri dishes. Triplicate aliquots of 100 μL of each treatment described above, individually combined with the synthetic germination stimulant GR24 10^−6^ M were applied to GFFP discs. Treated seeds were incubated in the dark at 23 °C for 7 days and radicle growth and proportion of radicles that developed haustorium was determined for each GFFP disc using a stereoscopic microscope (Leica S9i, Leica Microsystems GmbH, Wetzlar, Germany).

### 3.8. Statistical Analysis 

Percentage data were approximated to normal frequency distribution by means of angular transformation (180/π × arcsine (sqrt[%/100]) and subjected to analysis of variance (ANOVA) using SPSS software for Windows, version 21.0 (SPSS Inc., Chicago, IL, USA). The significant of mean differences between each treatment against the negative control was evaluated by the two-sided Dunnett test. Null hypothesis was rejected at the level of 0.05.

## 4. Conclusions

This manuscript reported for the first time the allelopathic potential of buckwheat root exudates and the effect of quercetin, isolated from buckwheat root, and its natural analogues apigenin and 3-*O*-acetylpadmatin isolated from *L. stoechas* and *D. viscosa*, on radicle growth of *P. ramosa*. SAR correlations were observed and discussed highlighting the importance for the activity of the quinone/hydroquinone oxo-reductive couple to impart activity. Besides reduction of radicle growth, haustorium induction was observed at the tip of *P. ramosa* radicles which swelled and a layer of papillae was formed. An additional haustorium assay was performed to study the haustorium inducing activity of quercetin in comparison with 2,6-dimethoxy-*p*-benzoquinone and a three partial methyl ether derivatives semisynthetized by quercetin. Results indicated that *P. ramosa* haustorium was induced by 2,6-dimethoxy-*p*-benzoquinone at concentrations of 1–0.5 mM and quercetin and its derivatives at concentration range 0.1–0.05 mM. In particular, the presence of two *ortho*-free hydroxy groups of C ring, like catechol, could be an important feature to impart activity while the carbon skeleton of B ring and substituents of both A and B rings are not essential. However, other experiments are needed to further support that the oxo-reductive mechanism is involved in reduction of radicle growth and haustorium induction activities.

## Data Availability

The data presented in this study are available on request from the corresponding author.

## Supplementary Materials

The Supplementary Materials are available online at https://www.mdpi.com/2223-7747/10/3/543/s1.
