# Supplementary material for: Allelopathic Effect of Quercetin, a Flavonoid from Fagopyrum esculentum Roots in the Radicle Growth of Phelipanche ramosa: Quercetin Natural and Semisynthetic Analogues Were Used for a Structure-Activity Relationship Investigation
Source: Plants (Basel). 2021 Mar 13;10(3):543. doi: 10.3390/plants10030543 (PMC8001586; doi:10.3390/plants10030543)
Supplement: Supplementary file 1 [file plants-10-00543-s001.pdf]

Supporting Information

Allelopathic effect of quercetin, a flavonoid from *Fagopyrum esculentum* roots in the radicle growth of *Phelipanche ramosa*: quercetin natural and semisynthetic analogues were used for a structure-activity relationship investigation.

Monica Fernández-Aparicio [1,†,*], Marco Masi [2,†], Alessio Cimmino [2], Susana Villarino [3] and Antonio Evidente [2,*]

[1] Institute for Sustainable Agriculture, Spanish National Research Center, Cordoba, Spain; monica.fernandez@ias.csic.es (M.F-A.)
[2] Department of Chemical Sciences, University of Naples Federico II, Complesso Univ. Monte Sant'Angelo, Via Cintia, Napoli, Italy; marco.masi@unina.it (M.M.); alessio.cimmino@unina.it (A.C.) evidente@unina.it (A.E.)
[3] ALGOSUR S.A., Ctra Lebrija-Trebujena km 5.5, Lebrija – Sevilla, Spain; svilarino@algosur.com (S.V.)
* Correspondence: authors contributed equally.
* Correspondence: monica.fernandez@ias.csic.es; evidente@unina.it

Citation: Fernandez-Aparicio, M.; Masi, M.; Cimmino, A.; Villarino, S.; Evidente, A. Allelopa-thic effect of quercetin, a flavonoid from *Fagopyrum esculentum* roots in the radicle growth of *Phelipanche ramosa*: quercetin natural and semisynthetic analogues were used for a SAR investigation. *Plants* **2021**, *10*, 543. https://doi.org/10.3390/plants10030543

Received: date
Accepted: date
Published: date

Publisher's Note: MDPI stays neutral with regard to jurisdictional claims in published maps and institutional affiliations.

Copyright: © 2021 by the authors. Submitted for possible open access publication under the terms and conditions of the Creative Commons Attribution (CC BY) license (http://creativecommons.org/licenses/by/4.0/).

Identification of quercitin (1) and p-coumaric acid methyl ester (4)

The ESI MS of quercitin showed a pseudomolecular ion $[M + H]^+$ at *m/z* 303, while *p*-coumaric acid methyl ester, showed in the same spectrum the pseudomolecular ion $[M + H]^+$ at *m/z* 179

Identification of apigenin (2)

The ESI MS of apigenin showed the pseudomolecular ion $[M + H]^+$ at *m/z* 271.

Identification of 3-O-acetylpadmatin (3)

The ESI MS spectrum of 3-*O*-acetylpadmatin showed the dimmer sodiated $[2M + Na]^+$ and the sodium $[M + Na]^+$ adduct ions at *m/z* 743 and 361 .

Charaterization of methyl ethers of quercitin (6-8)

The ^{1}H NMR spectra of **6** and **7** were reported in Materials and Methods and SI. They are very similar themselves and differed from that of quercetin for the presence of the two singlets of the two methoxy groups observed at δ 3.96 and 3.95 (4'-OMe and 7-OMe) in **6** and 3.90 and 3.82 (7-OMe and 3-OMe) in **7**. Their ESI MS spectra showed the same sodium adduct ion $[M + Na]^+$ at *m/z* 353. The ^{1}H NMR spectrum of **8** differed from that of **1** essentially for the presence of three singlets due to four methoxy groups (two of them were overlapped) at δ 3.97, 3.88 and 3.87. Its ESI MS spectrum showed sodium adduct ion $[M + Na]^+$ at *m/z* 381.

Location of the methoxy group in derivatives (6-8) by NOESY NMR spectra

The location of the methoxy groups in the structure of derivative **6-8** was based on the correlations observed in their NOESY spectra. In fact, in the NOESY spectrum of **6**, the methoxy group at δ 3.96 at C-4' showed a correlation with H-5' while that at δ 3.95 at C-7 correlated with H-8. In the NOESY spectrum of **7**, the methoxy group at δ 3.90 at C-7 correlated with both H-6 and H-8, while the other at δ 3.82 as did not give any correlation and in particular with the proton of C-ring and was located at C-3 being the hydroxy group at C-5 involved in a hydrogen bond with the carbonyl group at C-4. These latter linkage also justified the isolation of the 3,7,3',4'-*O*,*O*',*O*'',*O*'''-tretramethyl derivative of quercetin (**8**) and not the pentamethyl derivative.
